# Homelessness in autistic women: Defining the research agenda

**DOI:** 10.1177/17455057221141291

**Published:** 2022-12-14

**Authors:** Georgia Lockwood Estrin, Victoria Aseervatham, Clara M De Barros, Tara Chapple, Alasdair Churchard, Monique Harper, Emily JH Jones, William Mandy, Victoria Milner, Sarah O’Brien, Atsushi Senju, Chloe Smith, Jonathan Smith

**Affiliations:** 1 Department of Psychological Sciences, Birkbeck, University of London, London, UK; 2 School of Psychology, University of East London, London, UK; 3 Westminster City Council, London, UK; 4 Independent Scholar, Epsom, UK; 5 Porchlight, London, UK; 6 Oxford Institute of Clinical Psychology Training and Research, Oxford, UK; 7 SHP Westminster Floating Support, London, UK; 8 Research Department of Clinical, Educational and Health Psychology, University College London, London, UK; 9 Institute of Psychiatry, Psychology & Neuroscience, King’s College London, London, UK; 10 Faculty of Nursing, Midwifery & Palliative Care, King’s College London, London, UK; 11 Research Center for Child Mental Development, Hamamatsu University School of Medicine, Hamamatsu, Japan; 12 West London Mission (WLM), London, UK

**Keywords:** autism, homelessness, research priority, service provision, social services, women

## Abstract

**Background:**

Current evidence suggests that autistic individuals are at high risk for becoming and remaining in a cycle of homelessness. Key risk factors for homelessness disproportionately affect autistic people; however, we have limited understanding of how to best support autistic individuals accessing services. This gap in the evidence base is particularly acute for autistic women.

**Objective:**

As a first step to address this gap, we aimed to (1) map gaps in knowledge and practice; (2) identify priority areas for research and (3) develop recommendations for how to implement novel research and practice in this area.

**Methods:**

We conducted a collaborative workshop with an interdisciplinary group of 26 stakeholders to address our aims. Stakeholders included autistic women with experience of homelessness, researchers, health professionals, NGO representatives, and service providers.

**Results and recommendations:**

Two research priority areas were identified to map the prevalence and demographics of autistic women experiencing homelessness, and to delineate risk and protective factors for homelessness. Priority areas for improving provision of support included staff training to improve communication, awareness of autism and building trust with service providers, and recommendations for practical provision of support by services.

**Conclusions:**

Future research is critical to increase our knowledge of the pathways leading to homelessness for autistic women, and barriers to engaging with homelessness and social services. We need to use this knowledge to develop new ways of delivering targeted and inclusive support for autistic women, which could prevent or shorten periods of homelessness.

## Introduction

Autism is a spectrum and impacts people in different ways; it is defined by NICE^1^ as a lifelong, neurodevelopmental condition characterized by differences in social interaction and communication, the presence of rigid and repetitive behaviour, and restricted interests. Recently, there has been a shift in the conceptualization of autism as a form of human difference, and not as a ‘disorder’ defined by deficit.^2^ By recognizing this, we do not undermine the potential impact autism-associated traits, including difficulty with cognitive and behavioural flexibility, altered sensory sensitivity, sensory processing difficulties and emotional regulation difficulties, can have on daily activities.^1^ Autism also often co-occurs with physical, developmental and mental health conditions.^3^ Therefore, autistic people may present with a range of complex needs.

Autistic people often experience a range of risk factors for homelessness. Unemployment^4^ and poverty^5^ are a major cause, and reported as a significant challenge for autistic adults; only approximately, a quarter of autistic people are in any type of employment according to the Office for National Statistics’^4^ recent figures. Relationship breakdown and lack of social support are also risk factors for homelessness, and, again, are disproportionately felt by autistic people, with 79% reporting feeling socially isolated.^6^ This evidence suggests that autistic people are more likely to experience homelessness, and indeed, of the limited data that exist, the prevalence of autism in homeless populations has been estimated to be more than 12%.^7^ Homelessness carries with it an increased risk of premature mortality compared to the general population, and increased prevalence of mental disorders and substance misuse.^5^ This population is therefore in particular need of accessible services. Despite this, there is limited research on the link between autism and homelessness, and how to best provide services that could prevent or shorten periods of homelessness in this population. This is particularly true for women, who are more likely than men to avoid homelessness services due to a lack of appropriate, physically safe facilities and support tailored for women.^11^

Emerging evidence suggests that autistic people are not only more likely to experience homelessness, but also that their homeless experiences may be different than non-autistic people.^7–10^ Autistic individuals may have fewer means of avoiding homelessness and can face particular challenges to resolving their homelessness; specifically accessing and engaging with homelessness services.^8^ A recent narrative study with 10 autistic adults (two women) highlighted that barriers to accessing services led to disengagement with services and thus perpetuated homelessness.^12^ Overall, we have very limited knowledge of the specific factors causing homelessness or keeping autistic individuals’ in the cycle of homelessness, especially for autistic women.

Homelessness is a broad term, for which there is no agreed consensus on the definition.^5^ It not only refers to people sleeping in the open air, in uninhabitable buildings or other places not intended for human habitation (often termed ‘rough sleeping’), but also includes individuals sleeping in unsuitable, or temporary accommodation, such as hostels, and people who do not have a legal title to their accommodation, or safe access to private spaces.^5,13^ Most research on homelessness has focused on rough sleeping, which may not capture ‘hidden homelessness’, which includes people without stable accommodation. Women represent a large proportion of this group, principally because women’s experiences of sleeping rough on the streets often include sexual exploitation, abuse, violence and stigmatization.^11,14^ As a result, women have been forgotten in research and practice, and most of our current understanding about homelessness may be more applicable to men than women.

There is therefore a critical need to understand the factors leading to homelessness for autistic women and to use this knowledge to develop new ways of delivering targeted and inclusive support. As an integral step towards this, we conducted a collaborative workshop that aimed to (1) map gaps in knowledge and practice; (2) identify priorities for research and (3) develop recommendations for how to implement novel research and practice in this area.

## Methods

To achieve our aims, we adopted principles of participatory research^15^ and designed two workshops around Freire’s^16^ framework for generating open dialogue and creative listening to generate new ideas; a framework that been used to bring together methods in inclusive research.^17^

### Community involvement

An interdisciplinary group of 26 stakeholders took part, of which 6 identified as autistic. Participant-researchers (referred to henceforth as attendees) were purposively sampled to ensure expertise and representation from a diverse range of stakeholders. Our inclusion criteria specifically included individuals with lived experience of homelessness, autistic representatives, researchers, homelessness and domestic violence charity representatives, service providers, health professionals and Commissioners (many attendees fitted into multiple categories). Some attendees were decision-makers operating wide services in the United Kingdom, and therefore represented expertise from a broad context. Five attendees had experience of homelessness, and three female attendees identified as being autistic and with personal experience of homelessness.

### Workshop design and setting recommendations

Workshops were facilitated by a group experienced in working with multi-disciplinary stakeholders. Prior to the workshop, all attendees were sent a ‘Briefing document’ (Supplementary File A), which outlined (1) the aims of the workshops (see above), (2) what attendees could expect from the workshop and (3) what to expect following the workshop. In addition, the Briefing document outlined the set of principles designed to encourage inclusivity and open, active discussion. Attendees emailed their agreement (consent) to these principles and workshop expectations prior to the workshops.

The workshops took place for 2 days; each day began with narratives from different stakeholders, followed by facilitated break-out discussion sessions, before a plenary. Each break-out group was purposely chosen to include a mix of disciplines. The focus of the first day was to identify gaps in knowledge and practice, and priority areas for research. Ideas were consolidated and a summary was fed-back to stakeholders for discussion at the start of the second workshop, after a day of individual reflection. At this discussion session, priorities based on the previous workshop were listed, and a discussion session allowed reflection on whether these were the agreed priorities. Additional discussion on these priorities was conducted through a written summary document following the workshops (see below). We then built on these discussions to develop a research agenda and recommendations for service provisions, by breaking into smaller groups to discuss each priority area, and feeding back to the whole group for reflection and final in-person discussion on each priority and recommendation (Supplementary File A).

Following the workshops, a summary document was written (by first author, G.L.E.) to consolidate discussions and outputs. All attendees were invited to reflect and comment. Any ensuing discussions regarding priorities and recommendations were facilitated via Google Docs and were discussed until consensus was reached among stakeholders.

## Results and recommendations for research

Two key areas of research priority were identified: (1) establishing the prevalence and demographics of autistic women experiencing homelessness; and (2) understanding the risk and protective factors of homelessness for autistic individuals (Table 1). The workshop highlighted the importance of research to be co-produced with autistic women and with individuals who had experience of homelessness. This should include the use of peer-conducted research to promote trust between researchers and participants, and to encourage active engagement and participation.^14^

**Table 1.** Recommendations for research.

Research	Recommendations	Details
Research accessibility and impact	Co-production of research	Including peer-led research and using person-centred data collection methods ^a^
Methodology	Mixed methodology – Triangulation of multiple approaches Multiple approach to sampling – to understand the full range of experiences	Quantitative and qualitative methods Recruitment from clinical (autism, mental health, GP surgeries), social, homelessness, prison, hospitals, domestic violence, and police services
Measurement	Improved measurement of female autism – Adaptation and development of tools	Tools to be inclusive of autistic traits relevant to female autism, for example, masking
Ethical considerations	Safeguarding Safe environment for conducting research	Support for homeless women with or without a clinical diagnosis of autism Safe environment, for example, women-only spaces, or individual’s own accommodation Flexible environment to minimize sensory overload (e.g. minimal noise, adjustable lighting, temperature control), and use of a mixture of approaches based on needs

GP: General Practitioners.

a Example guidelines for co-production can be found here: https://www.scie.org.uk/co-production/; https://www.autistica.org.uk/our-research/research-toolkit/good-practice.

### Prevalence and demographics of autistic women experiencing homelessness

We need accurate estimates of the prevalence and demographics of autistic women who are homeless. This includes both the prevalence of autistic women who become homeless, and the prevalence of homeless people who are autistic. This is necessary to first evidence the needs of this group for clinical and homelessness services, respectively, and second to place autism at the top of priorities for appropriate changes to be made within policy and practice.

Two main challenges were identified in conducting this research. The first is our current definition of homelessness. Most of our knowledge about homelessness captures individuals who are ‘rough sleeping’ but exclude the ‘hidden homeless’ population. This is a particular barrier for identifying women, who represent a large proportion of this group. Engaging autistic women in this research who may be ‘hidden’ requires working closely with health services, domestic violence services, homelessness services, food banks and online via social media. Homelessness services should specifically include women’s shelters, sheltered accommodation and housing services.

To address this gap, the first step is to collate individual definitions and create an agreed definition of homelessness, especially ‘hidden homelessness’ in women (e.g. The European Typology of Homelessness and Housing Exclusion (ETHOS) which was developed to facilitate a common definition of homelessness to promote improved research and policy on homelessness^5^). Comprehensive definitions are important to establish accurate statistics and to understand the impact of different forms of homelessness on individuals.

The second major challenge to this research is the barrier to obtaining a diagnosis of autism for women. Autistic women are underserved by the clinical criteria for autism diagnosis, and this is partially due to a misunderstanding of autism in females.^18,19^ Therefore, to ensure that research is inclusive of diverse experiences, research must include autistic women who may not have a clinical diagnosis. Due to the high proportion of autistic women diagnosed with, for example, borderline personality disorder, research sampling from mental health facilities, hospital admissions, GP surgeries, and from autism clinics would be beneficial. A trait-based approach to sampling would be beneficial, rather than a categorical diagnostic-based approach, to increase inclusivity in research for individuals without a formal diagnosis.

The final recommendation for research was to improve our understanding of female autism and to develop or adapt tools to examine the aspects of autism that may be more prevalent in women than men. Camouflaging, ‘a dynamic process by which autistic individuals modify their innate autistic social behaviour in order to adapt, cope within, and influence the predominantly neurotypical environment’^20^, has been increasingly documented, particularly in autistic women. It may be a factor in the under-recognition of autism in women.^18,21^ Camouflaging has been associated with greater risk of mental health problems,^21^ and individuals not receiving specialist services and adequate support. Without receiving such specialized support, autistic individuals may be at heightened risk of homelessness, and camouflaging may also perpetuate homelessness for autistic individuals when attempting to access homeless and housing services. Some measures have been developed (e.g. Camouflaging Autistic Traits Questionnaire (CAT-Q)); however, further development of these tools and evaluation of their potential use within services could improve identification and help to provide targeted support for autistic women.

#### Ethical considerations for research

As many autistic women experiencing homelessness may not have a clinical diagnosis, the need to approach the concept of an autism diagnosis sensitively and with appropriate support in place was highlighted, specifically considering associated stigma and potential trauma that may have been experienced by women in these circumstances. Some women may also not be aware that their living situation could be defined as ‘homeless’, and research needs to consider how this subject can be approached in a safe and sensitive way.

Due to the link between domestic violence and homelessness for women, research needs to ensure strong links with domestic violence support services. Research must also be conducted in a safe space, such as in female-centred environments, or in an individual’s own preferred setting or the exploration of the use of online research; individuals potentially preferring to engage online where they can be in control of their environment more effectively.^22^

Finally, it was highlighted that researchers need to put appropriate safeguarding measures in place and ensure that any tools developed to improve identification of autistic women would not be misused, for example, to exclude individuals from services.

### Factors driving homelessness for autistic women

We need to capture the full experience of homelessness for autistic individuals; this includes improving our understanding of factors contributing to becoming homeless, and then the cascades of consequences of becoming, and remaining, in the cycle of homelessness. This research will increase our understanding of the needs of autistic women from services. It was highlighted that women often take different trajectories through homelessness than men. These differences are important to recognize, and research is required to not only investigate which factors driving homelessness are specific to autism but also factors that are specific to gender, and its intersection with autism. Increasing our knowledge and understanding of these factors is necessary to help to tailor services to meet an individual’s needs.

Gender-based violence is a particularly strong risk factor for women experiencing homelessness. Women’s homelessness is closely associated with gender-based violence, and often homelessness is a direct result of domestic violence and abuse.^11,14^ While estimates vary, a recent report showed that 20% who have experienced violence become homeless, compared with just 1% of women who have not experienced violence.^23^ This risk may be enhanced for autistic women who are at heightened risk of abuse.^24^ Autistic women may therefore be particularly vulnerable to becoming homeless due to the need to escape victimization. However, this association needs further investigation, alongside developing targeted, trauma-informed support for autistic women.

Environmental factors (e.g. contributing to sensory overload) were identified as critical contributors to reduced service access by autistic individuals, and which are in need of research to understand their association with homelessness. These included burnout, masking, stigma, lack of trust for services (further detailed below), and their contribution to meltdowns or shutdown. Meltdowns are intense and exhausting experiences, and happen when someone becomes overwhelmed and temporarily loses behavioural control; this can be verbal loss of control (such as screaming, crying) or physical (e.g. kicking, biting) or both.^25^ Research into individuals’ experiences of homelessness will clarify the impact of services on autistic individuals, and illuminate where, why and how individuals lose trust in services, which could help identify key inflection points where additional support within services would provide maximum impact.

A mixed methodological approach is required, including both qualitative and quantitative methods. Experiential qualitative methodologies, such as grounded theory, interpretative phenomenological analysis and narrative analysis, may be particularly valuable. The workshop highlighted the importance of taking an intersectional approach to this research, to explicitly include experiences of autistic women from minoritized ethnicities, and experiences of LGBTQ + individuals, as these individuals may experience double disadvantage in barriers to and within services.

### Recommendations for provision of support from services

Two priority areas were identified for improving accessibility and provision of support for autistic women: (1) improving communication, awareness of autism and building trust with service providers; and (2) practical provision of support.

#### Communication, awareness and trust

Improving the attitude towards autistic women and providing an autism-friendly culture of organizations were considered integral to generate trust between services and individuals using that service. Recommendations included assessing organizations’ attitudes towards inclusion, and ensuring that there is a focus on compassion within services. A further recommendation was the use of ‘autism champions’ within services, with an individual leading on specific autism strategies within the organization. Any adaptation to service provision must be done in collaboration with autistic individuals.

Improving accessibility crucially involves providing autism training to service providers; training should be co-facilitated with an autistic trainer. Training should be conducted with the aim of increasing awareness about autism, changing attitudes and culture around autism, and facilitating engagement with services. Training needs to encourage a flexible, person-led and strength-based approach (see Table 2).

**Table 2.** Recommendations for practice.

Service recommendations	Details
Improving accessibility of services Improving the culture and attitude within organizations around autism Autism training for service providers Improve communication between services (e.g. health, accommodation, benefits etc.)	– Use of “autism champions” within organizations – Person-centred and holistic approach, which is strength-based and flexible – Increase staff knowledge, awareness and acceptance of autism – Creating trust and mutual understanding between service provider and individuals using services
Practical support ^*^	– Provide a safe space to meet – Provide a space to meet with minimal factors that might contribute to sensory overload (e.g. minimal noise, adjustable lighting, temperature control, opening windows, etc.) – Provide a mixture of in-person support, phone support, email support, based on preferences – Respect for sensory and information overload, and understanding of their potential consequences, for example, meltdown – Respect preferences for time of day to meet – Adapt communication style, including making use of imagery, pictures, media – Importance of reliability and predictability of support (e.g. meeting on time and fulfilling promises) – Co-develop social scripts – Provide questions in advance of meeting – Offer additional support for filling out forms – Offer simple supports, such as ear plug and sleeping masks – Offer flexibility for accommodation needs, for example, in meal times and food preferences, and control over environment to minimize sensory overload

* for further guidance from a video developed by co-authors, please see: https://youtu.be/L3HVSu527MU

#### Practical support

The workshop highlighted that services are not built with autistic people or women in mind, this includes specific needs for accommodation (e.g. control over the environment, such as noise, light, temperature, smell) and safe spaces. For autistic women, the need to provide women-centred homeless services that are comfortable and safe was emphasized. Practical suggestions are outlined in Table 2.

Finally, integrating services to meet the needs of an individual as a whole (a holistic approach) was recommended, rather than discrete elements, such as housing without addressing the underlying need for support, which would be supported by envisioning a long-term and preventive approach to homelessness.

## Conclusion

This workshop identified research priorities in the field of homelessness and autism in women, and recommended areas for improving accessibility and engagement with services. Conducting this research is critical to increase our knowledge of the pathways leading to homelessness for autistic women, and barriers to accessing services. We need to use this knowledge to develop new ways of delivering targeted and inclusive support for autistic women, which could prevent or shorten periods of homelessness.

## Supplemental Material

sj-docx-1-whe-10.1177_17455057221141291 – Supplemental material for Homelessness in autistic women: Defining the research agenda Click here for additional data file. Supplemental material, sj-docx-1-whe-10.1177_17455057221141291 for Homelessness in autistic women: Defining the research agenda by Georgia Lockwood Estrin, Victoria Aseervatham, Clara M De Barros, Tara Chapple, Alasdair Churchard, Monique Harper, Emily JH Jones, William Mandy, Victoria Milner, Sarah O’Brien, Atsushi Senju, Chloe Smith and Jonathan Smith in Women’s Health

## Declarations

**Ethics approval and consent to participate:** This project was approved by the Ethical Committee of the Department of Psychological Sciences, Birkbeck, University of London (Approval No. 2021081).

**Consent for publication:** Not Applicable.

**Availability of data and materials:** Due to the nature of this research, participants of this study did not agree for their data to be shared publicly, so supporting data are not available.
