# Supplementary material for: Homelessness in autistic women: Defining the research agenda
Source: Womens Health (Lond). 2022 Dec 14;18:17455057221141291. doi: 10.1177/17455057221141291 (PMC9756367; doi:10.1177/17455057221141291)
Supplement: sj-docx-1-whe-10.1177_17455057221141291 – Supplemental material for Homelessness in autistic women: Defining the research agenda [file sj-docx-1-whe-10.1177_17455057221141291.docx]

Autism and homelessness in women

**Briefing Booklet contents**

##

**Logistics**

Workshop attendance

Accessibility

Chatham House Rules

Protocols for Online Video Workshop

**Information generated and recording of the workshop**

Recording

What will you do with the recorded information from this workshop?

**What to expect from this workshop**

Why is this important?

Workshop Aims and Outputs

Rough Agenda

**What to expect following this workshop**

## **Logistics**

**Workshop Attendance**

**Accessibility**: We are keen to make this workshop, and any further project activities, as accessible as possible for all individuals to fully participate. If you have any suggestions or have particular queries and/or requirements, please get in touch.

**Chatham House Rules**: The workshop will be run under the Chatham House Rule to facilitate open communication: “When a meeting, or part thereof, is held under the Chatham House Rule, attendees are free to use the information received, but neither the identity nor the affiliation of the speaker(s), nor that of any other attendees, may be revealed.” Therefore, nothing that is said during the workshop will be attributed to any individual in any of the documents proceeding the workshop or in any oral discussions/presentations about the workshop.

**Protocols for the Online Workshop**: When you join the meeting, your microphone will be muted and your video will be set to off. We invite you to turn on your video once you are ready, and we ask that you keep your microphone muted except for when you are speaking. This will keep background noise to a minimum.

We may discuss some topics during the workshop that are sensitive. We will provide a “Quiet room” online or invite participants to take a “time-out” if needed. The chat function on the zoom platform can also be used to voice any concerns with the facilitator or researchers, if they arise.

## **Information generated and recording of the workshop**

**Recording**: Please note that the workshop will be audio/video recorded, and notes will be taken. The recordings and notes will remain internal, and will not be released beyond the researchers. It will serve to help ensure accuracy and thoroughness in summarizing the discussion.

### **What will we do with the recorded information from this workshop?**

We will make every effort to ensure that all the information discussed in the workshop will remain confidential, as per the Chatham House Rules described above. The recording of this workshop and notes will be kept in a password protected file on a secure computer and securely backed up, and will only be accessible by the immediate researchers of this project. This information will be destroyed after ten years following project completion.

The workshop has received ethical approval from the XXX.

**What to expect from this workshop**

The aim of this workshop is to bring together a diverse group of individuals to discuss and identify priority areas of research relating to autism and homelessness.

**Workshop Aims and Outputs**

In this workshop, we aim to:

- understand where there are gaps in our knowledge and practice in autism and homelessness in women

- Discuss priority areas for research focus

- Develop research questions

- Discuss and develop best practice of how to answer these research questions

## **Rough Agenda**

| **WORKSHOP 1: October 12 2021** | |
| --- | --- |
| **09.45** | **Waiting Room opens** |
| **10.00** | **Welcome and introductions** |
|  | **Introduction and Context**  **Followed by Insight talks** |
|  | **Breakout discussions, feedback and plenary** |
|  | **Insight talks from** |
|  | **Breakout discussions, feedback and plenary** |
|  | **Closing comments and introduction to workshop 2** |
| **12.00** | **CLOSE** |

| **WORKSHOP 2 : October 14 2021** | |
| --- | --- |
| **09.45** | **Waiting Room opens** |
| **10.00** | **Welcome to Workshop 2** |
|  | **Networking** |
|  | **Overview of Workshop 1**  **Followed by Insight Talks** |
|  | **Breakout discussions , feedback and plenary** |
|  | **Short plenary** |
|  | **Closing thoughts reflections** |
| **12.00** | **Next steps** |

## **What to expect following this workshop**

Following this workshop, a written summary document of the workshop proceedings will be sent to all attendees. This will be based on the notes taken during the workshop, and from the recording of the workshop. Attendees will have the opportunity to read over and comment on this summary.

## 
